# Teaching Residents Chest Tubes: Simulation Task Trainer or Cadaver Model?

**DOI:** 10.1155/2018/9179042

**Published:** 2018-07-24

**Authors:** Ting Xu Tan, Paula Buchanan, Erin Quattromani

**Affiliations:** ^1^Department of Surgery, Division of Emergency Medicine, Saint Louis University School of Medicine, 3635 Vista Ave, St. Louis, Missouri, 63110, USA; ^2^Center for Health Outcomes Research, Saint Louis University, 3545 Lafayette Ave, Room 409B, St. Louis, MO 63108, USA

## Abstract

**Objective:**

To compare simulation task trainers (sim) with cadaver for teaching chest tube insertion to junior residents.

**Methods:**

Prospective study involving postgraduate year (PGY) one and two emergency medicine (EM) and PGY-1 surgery residents. Residents were randomized into sim or cadaver groups based on prior experience and trained using deliberate practice. Primary outcomes were confidence in placing a chest tube and ability to place a chest tube in a clinical setting during a seven-month follow-up period. Secondary outcomes include skill retention, using an objective assessment checklist of 15 critical steps in chest tube placement, and confidence after seven months.

**Results:**

Sixteen residents were randomized to cadaver (n=8) and simulation (n=8) groups. Both groups posttraining had statistically significant increase in confidence. No significant difference existed between groups for median posttraining assessment scores (13.5 sim v 15 cadaver). There was no statistically significant difference between groups for confidence at any point measured. There was moderate correlation (0.58) between number of clinical attempts reported in a seven-month follow-up period and final assessment score.

**Conclusion:**

Both sim and cadaver models are effective modalities for teaching chest tube placement. Medical education programs can use either modalities to train learners without notable differences in confidence.

## 1. Introduction

Emergency medicine (EM) and surgery residents are expected to become skilled and confident in placing chest tubes. The Accreditation Council of Graduate Medical Education (ACGME) classifies chest tubes (tube thoracostomy) as a high frequency and high-risk procedure, requiring EM residents to complete a minimum of ten chest tube placements during residency and surgical residents to be directly supervised until competency is demonstrated [1–3]. The council, however, does not provide guidelines for ensuring procedural competency. There is also no standardized educational method for teaching this procedure.

Of note, the chest tube insertion technique discussed and taught in this study is distinct from the Seldinger technique typically used to place pigtail catheters to drain pneumothoraces and small effusions in clinically stable patients [4]. Briefly, the Seldinger technique involves insertion of a needle into the pleural space and advancement of a guidewire through the needle followed by dilation of the insertion site. The pigtail catheter is then inserted over the guidewire into the pleural space [5]. This is in contrast to the standard tube thoracotomy or chest tube placement involving a skin incision at the anterior axillary line at the fifth intercostal space, followed by blunt dissection through muscle and above the rib, and into the pleural space. A chest tube is then inserted through the dissected pathway into the pleural space [6].

Medical simulation has emerged as a major educational tool for developing procedural expertise [1]. Mannequin task trainer and animal and cadaver models have all been utilized to teach tube thoracostomy with positive outcomes [7–12]. There are, however, few studies comparing different modalities for teaching chest tube placement: their outcomes are also limited to educational effects in a classroom or simulation laboratory.

This study examines the efficacy of simulation and cadaver models in teaching tube thoracostomy and the effect on a resident's clinical success. We hope this study will expand our understanding of the best educational methods to invest potential resources for teaching procedural competence to residents.

## 2. Materials and Methods

### 2.1. Study Design and Participants

This was a prospective randomized study at a single tertiary-care teaching hospital conducted from June 2015 to February 2016. Eight EM residents (PGY-1 and PGY-2) and eight general surgery residents (PGY-1) were recruited. All participants were deidentified and given a study ID. The governing institutional review board at Saint Louis University where the study was conducted determined that this study was exempt because of research conducted in commonly accepted educational settings.

### 2.2. Study Protocol

*Randomization. *Residents were divided into two learner groups based on total number of chest tubes placed in patients, simulators, cadavers, and/or animal models prior to the study. More experienced learners placed at least four chest tubes previously. Members within each group (more versus less experienced learners) were then randomized using a random number generator into either sim or cadaver group.

*Initial Training Session. *Prior to the procedural skill session, participants were emailed resources about the procedure to review independently. These resources included a New English Journal of Medicine (NEJM) chest tube insertion video [4] and a book chapter on pulmonary trauma from a widely used EM resource [6].

In July, residents participated in a hands-on deliberate practice training session according to their assigned modality. Training was conducted individually with a single trained faculty member (EQ). Participants were coached through an objective assessment checklist of 15 critical steps in chest tube insertion. This checklist was used in a prior chest tube study [13] and was minimally modified to include the final step of securing the tube, Table 1. Emphasis was placed on mastery of each step before proceeding to the next one. There were no time limits. Training was complete when the resident was independently able to carry out each step. The cadaver training group practiced with embalmed cadavers while the simulator group practiced on a TraumaMan simulator (SIMULAB Corporation, Seattle, WA).

*Final Assessment. *In February 2016, residents were assessed on their ability to place a chest tube using the same assigned training modality in July. The same faculty member (EQ) evaluated each resident using the 15 critical steps assessment checklist. No guidance or feedback was given until completion of the procedure.

### 2.3. Measurements and Key Outcomes

Prior to training, residents filled out a questionnaire assessing their perceived ability in placing a chest tube independently, where “independently” meant without hands-on assistance from a supervising clinician. They were also asked to identify confidence level in placing a chest tube pre- and posttraining (on a 10-point Likert scale with 1 “not confident” and 10 “most confident”). These questionnaires and all following were completed using Qualtrics TM, an Internet based survey program.

After training, participants logged each chest tube placed clinically before final assessment in February 2016. Information collected included month of procedure, general setting of procedure (such as Emergency Department, Intensive Care Unit, Inpatient Floor), ability to place a chest tube without hands-on assistance, whether the resident needed assistance with puncturing pleura, number of attempts required for the chest tube, whether the chest tube required additional chest tubes, or if any modification of chest tube placement was necessary after imaging with chest X-ray or CT scan. Finally, residents rated their confidence when placing that chest tube on a 10-point Likert scale.

At the final assessment session, residents were evaluated on a 15-point critical steps assessment checklist. Residents subsequently rated their final level of confidence in placing a chest tube (on a 10-point Likert scale).

Primary outcomes were confidence in placing a chest tube and ability to subsequently place a chest tube independently in the clinical setting during the study period after initial training. Secondary outcomes were retention of chest tube placement skills, as measured on their assessment scores, and confidence in their ability to place a chest tube at conclusion of the study.

### 2.4. Data Analysis

Data was collected in a computerized database (Microsoft Excel, Microsoft Corp, Redmond, WA). Data analysis was conducted with IBM SPSS v23 (IBM Corp, Armonk, NY). Participant characteristics and results were analyzed using descriptive statistics, including counts and percent for categorical data, and median and interquartile ranges (IQR) for continuous and ordinal data. Changes in scores from pre to post were calculated and compared between sim and cadaver groups using Mann–Whitney* U* tests. Wilcoxon Signed Rank test was utilized to determine if there was a change within a group from pre to post study period. Correlations were calculated using Spearman's rho test for ordinal variables and Fisher's exact test for categorical variables. Significance level was set at p < 0.05 for all analysis and only the first attempt was utilized for statistical comparisons.

## 3. Results

### 3.1. Characteristics of Study Participants

Twenty-two residents (10 general surgery and 12 EM) were recruited for the study. Sixteen residents (8 general surgery and 8 EM) agreed to participate. There were eight PGY-1 general surgery residents, five PGY-1 EM residents, and three PGY-2 EM residents. Eight residents were assigned to simulation and eight were assigned to the cadaver group based on prior experience in placing chest tubes. There were no significant differences in characteristics between the two groups in terms of age, prior experience or confidence in placing a chest tube prior to the training session. See Table 2.

All residents completed their assigned training session in July 2015, which lasted approximately one hour per resident. All residents completed their follow-up posttraining questionnaires for the subsequent seven months. From July 2015 to February 2016, residents placed a total of 42 chest tubes in a clinical setting (17 simulation task trainer and 25 cadaver group). See Table 3. Two residents did not place any chest tubes during this seven-month period (one in each group). All participating residents completed their final training assessment in February of 2016.

### 3.2. Primary Outcomes

Median (IQR) baseline confidence levels were low for both groups and not statistically different, 2.00 (0.25 – 6.25) (simulation) v 2.50 (0.5 – 4.0) (cadaver), p = 1.00. After training, both sim and cadaver groups had a statistically significant increase in median confidence to 8.00 (7.25 – 9.5) (p =0.01) sim and 8.00 (7.00 – 9.00) (p = 0.008) cadaver without statistical difference between groups (p = 0.88). See Table 4.

Thirteen of the fourteen residents who placed a chest tube in the clinical setting reported doing so without hands-on assistance by a supervisor. There was no statistical difference in ability to place a chest tube independently on first clinical attempt after training between residents in the simulation task trainer (57%) and cadaver group (71%), p = 1.00. Median number of days from training to first clinical attempt was 111 days (74 – 122.5) sim and 101 days (65 – 125.5) cadaver.

### 3.3. Secondary Outcomes

In the final assessment, retention of skill measured using the 15 critical step assessment checklist was similar in both groups. There was no significant difference between median (IQR) checklist scores (13.5 (11.25 – 15.0) sim v 15 (15.0 – 15.0) cadaver), p = 0.130. After final assessment, confidence remained high and similar in both groups (8 (7 – 9) sim vs 8.5 (7.25 – 9) cadaver), p = 0.72. There was no statistical difference between groups for confidence at any point measured during the study. There was moderate correlation between overall number of clinical attempts during study period and poststudy confidence (rho = 0.54, p = 0.36). Additionally, there was moderate correlation (rho 0.56, p = 0.025) between number of clinical attempts during the study period and final checklist score.

## 4. Discussion

Our study demonstrates that both simulation and cadaver models are effective teaching modalities for chest tube placement when utilized with deliberate practice. There was no control group of the traditional apprenticeship model since prior studies have already established that simulation-based education has significant effects on skills and patient outcomes compared to no intervention [14]. We also used a deliberate practice teaching model since mastery learning and feedback improve learning when compared with other forms of simulation-based education [14, 15]. Residents in both groups showed a statistically significant increase in confidence in their chest tube skills posttraining. This confidence remained high over the course of at least 7 months. These findings are consistent with previous studies demonstrating effectiveness of individual simulation modalities (sim and cadaver models) at increasing learner confidence and teaching procedural skill [9–12].

This is the first study we are aware of comparing efficacy of simulation task trainers with cadaver model in teaching tube thoracostomy to junior residents. Prior studies comparing animal models to simulation task trainers did not discern any statistically significant differences between the two modalities, particularly procedural outcomes, success rate, and perceived educational effectiveness [12, 16]. These studies were also limited to the educational or laboratory setting. Our goal was to go a step further to investigate translational outcomes of our educational intervention. Our results did not detect any statistically significant differences in confidence or ability to place a chest tube independently in a clinical setting following the training session on either cadaver or simulation task trainer. Of the 14 residents who placed a chest tube clinically, 13 (93%) indicated they were able to place a chest tube independently, without assistance from their supervising clinician. This finding is consistent with the clinical success rate from a study involving simulation-based deliberate practice on a simulator model for lumbar punctures where pediatric residents had a 94% clinical success rate on achieving CSF after the intervention training session [17].

When evaluating retention of skill and confidence at the follow-up session, there was no significant difference between groups in terms of checklist score or final confidence level. What seemed to matter, with a moderate degree of correlation, was number of chest tubes the resident placed in a clinical setting during the study period and their final checklist score. While this data shows that skills decay can be seen seven months after training, whether this correlation is clinically significant is unknown. There is currently limited data on EM and surgical resident chest tube placement clinical skills decay and training frequency required to maintain competency. A prior study has shown that pediatric resident chest tube placement skills decay can be seen as early as one month posttraining [18]. Future studies could explore optimal timing and minimal number of chest tubes performed to trigger a “refresher” sessions to minimize skill decay and optimize clinical success.

Like many studies on simulation-based training, this study is limited by small sample size at a single institution. Resident self-reported data in the clinical setting was also subjected to recall bias of confidence level and interpretation of “independently” placing a chest tube. Additionally, assessment of skill retention was done by a single unblinded faculty member.

Future studies would ideally include an assessment from the supervisor at the time of the bedside or laboratory procedure who was blinded to the resident's training group. We believe the use of a previously used checklist in the final assessment helped minimize bias in assessing the resident's performance. There was also no formal evaluation of the resident's competency outside the study to motivate residents to exaggerate their confidence or abilities when self-reporting.

## 5. Conclusion

Both educational methods of simulation and cadaver model for teaching chest tube placement are associated with significantly increased confidence of the resident. This study is still relevant to educational training programs because determining the best simulation-based modality for procedures has not been established. From our results, it does not appear the learner will have better or worse outcomes if the program decides to use cadaver or simulation-based learning for teaching chest tubes.

## Figures and Tables

**Table 1:** Objective assessment checklist of critical steps in chest tube insertion.

**Critical Element**	**Criteria Met**	**Criteria Not Met**
1. Identify insertion site 5th intercostal anterior mid-axillary line on affected side* or nipple line (not in females)*		

2. Sterile preparation of chest		

3. Anesthetize skin and subcutaneous tissue and periosteum of underlying rib and pleura just past the rib		

4. 2-3 cm incision, transverse incision parallel to the line of ribs, at the predetermined site with a 11 blade scalpel		

5. Bluntly dissect the subcutaneous tissue with a hemostat or scissors		

6. Dissect over the top of the underlying rib to the next highest intercostal space		

7. With the tip of the hemostat puncture the parietal pleura while pushing at the top border of the rib		

8. Enter the pleural space over the top of the rib to avoid damaging the neural vascular bundle		

9. Enter in a controlled fashion to avoid laceration to the lung		

10. Once inside the pleura, spread the hemostat widely and withdraw while still open		

11. Create a sufficient opening in the pleura for the chest tube		

12. Place finger inside hole of pleura and move finger 360 degrees to confirm correct location and assure no impediment and adhesions		

13. Clamp distal end of the chest tube (proximal optional)		

14. Connect to an underwater seal collection chamber		

15. Secure chest tube		

Total Points		

**Table 2:** Participant demographics and experiences.

	Simulation task trainer (n=8)		Cadaver model (n=8)	
EM residents	4	(50%)	4	(50%)

Surgery residents	4	(50%)	4	(50%)

PGY-1	6	(75%)	7	(87.5%)

PGY-2	2	(25%)	1	(12.5)

Male	5	(62.5%)	6	(75%)

Female	3	(37.5%)	2	(25%)

Median (IQR) age	27.5	(26 - 32)	28	(26 – 29.75)

Median (IQR) pre-study patients	0	(0 – 3)	0	(0 - 1)

Median (IQR) pre-study simulation task trainers	0	(0 – 1.75)	0.5	(0 – 3.75)

Median (IQR) pre-study cadavers	0.5	(0 – 2.75)	1	(0 – 1.75)

Median (IQR) pre-study animal models	0	(NA)	0	(0 - 0)

Independent	2	(25%)	1	(12.5%)

Not independent	6	(75%)	7	(87.5%)

**Table 3:** Clinical attempts.

	Simulation Task Trainer (n = 8)	Cadaver Model (n = 8)
Total number of chest tubes	17	25

Median (IQR) number of chest tubes placed clinically	2 (2 – 2.75)	3 (1.25 – 5.5)

Total number of chest tubes placed without hands-on assistance	12 (71%)	18 (72%)

Total number of chest tubes placed requiring assistance with puncturing pleura	3 (18%)	4 (16%)

Total number of chest tubes requiring additional chest tube	2 (12%)	2 (8%)

Total number of chest tubes requiring modification after X-ray or CT scan	2 (12%)	2 (8%)

ED	5	10

ICU	7	10

Inpatient floor	1	3

OR	3	1

Other location	1	1

**Table 4:** Confidence levels.

	Simulation task trainer (n=8) Median (IQR)		Cadaver model (n=8) Median (IQR)	
Pre-study confidence	2	(0.25 -6.25)	2.5	(0.5 – 4.0)

Post training confidence	8	(7.25 – 9.5)	8	(7 – 9)

Post-study confidence	8	(7 – 9)	8.5	(7.25 - 9)

Final assessment checklist score	13.5	(11.25 – 15)	15	(15 – 15)
